# Factors Associated With the Use of Digital Health and Well-Being Resources in Non–Memory-Led Dementias: Quantitative Survey Study

**DOI:** 10.2196/85863

**Published:** 2026-03-30

**Authors:** Emilie V Brotherhood, Céline El Baou, Caroline Fearn, Oliver Hayes, Nikki Zimmermann, Anastasia Tsipa, Sebastian J Crutch, Joshua Stott

**Affiliations:** 1 Dementia Research Centre, Queen Square Institute of Neurology, University College London, 8-11 Queen Square, London, WC1N 3AR, United Kingdom, +44 2034483666; 2 Division of Psychology and Language Sciences, University College London, London, United Kingdom

**Keywords:** mobile health, telemedicine, telehealth, telemonitoring, dementia, atypical Alzheimer disease, frontotemporal dementia, primary progressive aphasia, gerontechnology, non–memory-led dementia, young-onset dementia, posterior cortical atrophy

## Abstract

**Background:**

Digital platforms disseminating health information and providing support for the experience of non-memory-led dementias (NMLDs) are invaluable. However, the factors influencing engagement with these resources in people affected by NMLDs are poorly understood. We conducted the world’s largest survey exploring the experience of digital access in NMLDs to learn directly from people with a diagnosis, their care partners, and NMLD health care professionals (HCPs).

**Objective:**

This study aimed to determine factors associated with digital health and well-being resource use in people with NMLD and their care partners and investigate differences in digital health and well-being resource use according to NMLD subtype.

**Methods:**

Individuals diagnosed with NMLD—for example, those with frontotemporal lobar degeneration, posterior cortical atrophy (PCA), and primary progressive aphasia (PPA)—their care partners, and NMLD HCPs (N=450) responded to the survey. A subset of care partners provided two responses (carer-related and proxy), generating 4 survey groups with responses (N=538). The survey included demographics and basic clinical information, the outcome measure of usage behavior, and factors including constructs from the Short Senior Technology Acceptance Model (Short STAM) and additional constructs informed by a combination of previous literature and consultation with NMLD experts-by-experience (anxiety and depression [Patient Health Questionnaire–4-item], instrumental activities of daily living, web-related privacy and security concerns, and digital health literacy). Separate multiple linear regressions were run for each survey group to elucidate which variables predicted higher use of digital health and well-being resources. The use of digital resources for health and well-being was also explored across 3 NMLD subtypes: frontotemporal dementia (FTD), PPA, and PCA.

**Results:**

Attitudinal belief was consistently the strongest predictor of digital health and well-being resource use in people with NMLD as well as caregivers and proxy respondents. Control belief was significantly associated with higher digital health and well-being resource use in the NMLD and proxy groups; a trend was observed in the carer group. The adjusted *R*² range (63.0%‐70.8%) denotes the variance in digital health and well-being resource use accounted for in the 3 models. Lower digital health and well-being resource use was associated with FTD diagnosis and caregiver groups relative to PPA and PCA.

**Conclusions:**

Collectively, these findings indicate several factors are critical to consider when designing digital health and well-being resources for people with NMLD and their caregivers, in particular targeting practical and emotional perceptions of digital resource use for health and well-being. This should be undertaken in combination with design considerations that address condition-specific cognitive profiles encountered by those living with NMLD diagnoses and those who care for them.

## Introduction

### Background

Dementia is an umbrella term encompassing several diseases that cause progressive cognitive and functional decline, affecting over 57 million people worldwide, with 10 million new cases diagnosed each year [1]. While pharmacological therapeutic interventions are available for some forms of dementia, access remains inconsistent, and many individuals living with a dementia diagnosis—as well as their caregivers—rely heavily on psychosocial support and community-based services. eHealth and digitally delivered services designed for people affected by dementia are both an appealing way to disseminate relevant information and provide virtual opportunities for dementia care in the context of stretched resources (thus reducing reliance on geographical proximity to in-person services). The COVID-19 pandemic catalyzed a rapid shift toward digital health and well-being tools, accelerating the transition from paper-based and in-person community support provision to virtual platforms [2]. This shift opened numerous possibilities for dementia care, including increasing accessibility to dementia-related support groups [3]. Emerging evidence suggests that individuals with dementia and their caregivers engage with technology in similar ways, indicating that both audiences benefit from digital health and well-being provision [4].

The most common form of dementia is Alzheimer disease, with the majority of individuals presenting with episodic memory impairment as an early symptom [5]. However, a substantial proportion of people with dementia present with initial symptoms other than memory complaints. For example, individuals with an atypical variant of Alzheimer disease, posterior cortical atrophy (PCA), exhibit early visual dysfunction [6]. Changes in behavior and personality are characteristic of behavioral variant frontotemporal dementia (bvFTD [7]); language production and comprehension are a hallmark of the primary progressive aphasias (PPA [8]); and sudden-onset mood disorders are often accompanied by hallucinations in dementia with Lewy bodies [9]. These atypical presentations, collectively referred to as non–memory-led dementias (NMLDs), pose unique challenges for individuals with a diagnosis and their caregivers. In addition, symptoms often emerge before the age of 65 years (considered a young-onset dementia) with consequent implications for finances, work, and family [10]. The relative rarity of these conditions contributes to limited postdiagnostic service provision and low primary care professional and public awareness [11], while their geographical dispersion further complicates access to relevant face-to-face services [12]. Many of these challenges can be mitigated through virtual platforms, which can enable expert-led interventions [13] and peer support across geographical regions [14]. Capacity for digital engagement is high in people with NMLD as well as caregivers, owing to the fact that many affected individuals are younger than 65 years; in 2019, 73.9% of individuals aged between 50 and 64 years in England used the internet every day [15].

While digital services are a promising platform to meet the specific needs and reach of people affected by NMLDs, the factors influencing engagement with digital health and well-being resources—specifically among people living with NMLDs and their caregivers—remain underexplored, representing a critical gap in the literature. While digital platforms (eg, websites and videoconferencing) offer promising avenues for support, their usability may be directly affected by specific clinical and neuropsychological profiles associated with NMLDs. For example, anosognosia (lack of insight), which is characteristic of bvFTD, may influence likely engagement with condition-specific health and well-being materials if the individuals do not perceive themselves as having a disease. NMLD caregivers, too, face distinct potential barriers to digital health and well-being engagement. These include limited time to source and access digital health and well-being information due to demanding caregiving roles, often compounded by a lack of recognition or support from professionals and the broader public; insufficient privacy to participate in virtual support groups; and the emotional strain of navigating complex and frequently misunderstood symptoms. Understanding the specific factors associated with NMLD digital health and well-being resource use will optimize NMLD-specific digital resource and intervention design, maximizing subsequent engagement and uptake from the audiences with NMLD they aim to support.

**Figure 1.**
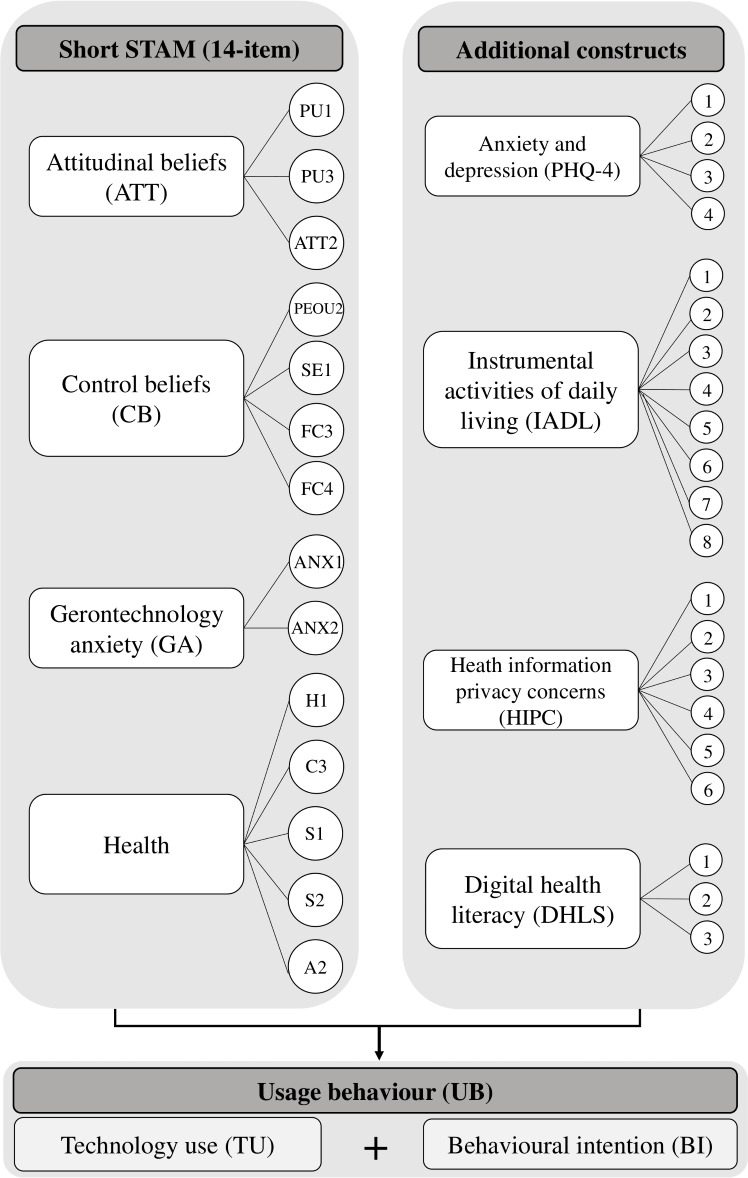
Construct of a map detailing the domains within the digital access survey. A: attitude toward aging; ATT: attitude toward using technology; C: cognitive ability; H: self-reported health conditions; FC: facilitating conditions; PEOU: perceived ease of use; PU: perceived usefulness; S: social relationships; SE: gerontechnology self-efficacy.

Previous models explaining variance in technology use and behavioral intention to use technology have sought to do so in a consumer context, such as the Unified Theory of Acceptance and Use of Technology (UTAUT [16]), the revised version of which explains up to 74% of behavioral intention to use technology and 52% of actual use [17]. Combining this with another predominant model for workplace technology use (Technology Acceptance Model [18]), Chen and Chan [19] developed a 38-item seniors-focused model to understand gerontechnological acceptance by older people from Hong Kong. Alongside original constructs pertaining to attitudinal beliefs and control aspects, Chen and Chan [19] included additional constructs of age-related health and independent capabilities, revised in a shortened version of the Short Senior Technology Acceptance Model (14-item; Short STAM), which demonstrated its accuracy in relation to assessing the propensity of individuals older than 55 years to engage with new technology, appropriate for dementia populations with a lower age of onset and their caregivers [20]. Low variance for the Short STAM (14-item) explaining technology usage behavior in subsequent studies [21] indicates other population-specific factors that may be important to consider when examining factors contributing to technology-use behavior in NLMDs. Qualitative work focusing on technology use in older adults with hypertension, for example, highlights other factors potentially influencing engagement, such as privacy concerns [22]. This factor may even be arguably more pertinent for studies exploring technology usage behavior in dementia populations, owing to acknowledged vulnerability to financial and technological scams and for people with NMLDs in the necessity to divulge sensitive category data by virtue of explaining symptoms (eg, a person living with posterior cortical atrophy disclosing their clinical phenotype to allow for reasonable adjustments to be made). Additional aspects influencing digital health and well-being resource use in people with NMLDs may include factors related to major neurocognitive changes (eg, changes in instrumental activities of daily living), shared experiences between people with dementia and their caregivers (eg, anxiety and depression), and more recently identified constructs such as digital health literacy that were not included in the Short STAM (14-item) model. Informed by these current models of technology use, we conducted the first quantitative study to our knowledge exploring these and additional constructs in people living with NMLDs and their caregivers (see Figure 1). We included subconstructs and item numbers from the Short STAM, the selection for which was derived from a sequential item-reduction strategy and confirmatory factor analysis of prior models (original STAM–38 items [19]). Standardized parameter estimates for interconstruct correlations for the Short STAM (14-item) are reported in full in the study by Chen and Lou [20]. The right-hand block indicates additional constructs added to the digital access survey in this study, with variable selection guided by technology-use investigations in other clinical cohorts [22] and patient and public involvement (PPI) consultation with NMLD experts-by-experience. Aligned with our focus on flexible health care provision, we focused on technology use relating to digital platforms providing resources in the domains of health and well-being (eg, websites, Zoom [Zoom Video Communications]) and remained agnostic to mode of access (eg, whether accessed via smartphone, tablet, or computer).

### Study Objectives

To address this gap, the following research questions were formulated:

1. What are the factors associated with digital health and well-being resource use in people with NMLD and their care partners?
2. Is there a difference in digital health and well-being resource use according to NMLD subtype?

## Methods

### Participant Characterization

Background demographic variables previously reported to influence usage (eg, age in years, gender, and ethnicity [17,23]) were collected alongside clinical characteristics such as NMLD type (eg, primary progressive aphasia, posterior cortical atrophy, and frontotemporal dementia), time since first symptoms, and formal dementia diagnosis.

The final sample comprised 450 participants with 538 survey responses, collected between March 2023 and March 2024. This exceeded our initial power calculations for analysis of participation issues affecting people with NMLD, which was computed at n=385 (assuming 10 predictors; power=90%; *R*^2^=0.33; α=.05). Taking a conservative approach, the minimum sample size for the 10 candidate predictor variables would be 385 (assuming outcome prevalence=50%, margin of error in intercept estimation =5%, 5% acceptable difference in apparent, and adjusted *R*^2^ of 0.2). Survey response N exceeds sample size owing to a group of carers providing 2 responses, one relating to their own experiences and proxy responses for the person with NMLD. Participants included 63 people with a diagnosis of NMLD, 269 carer reporting on their own digital health and well-being resource use, 105 proxy responses, and 101 health care professionals (HCPs).

### Survey Design and Measure Selection

The study was based on a cross-sectional questionnaire, using a participant-report approach. The survey was carefully designed to consider ease of administration and reduce participant burden where possible, in consultation with HCPs experienced in working with people with NMLD as well as caregivers and proxy respondents (eg, consultant neurologists, clinical psychologists, academic researchers, and support workers), and the Rare Dementia Support PPI group, comprising people living with and alongside NMLDs.

The theoretical constructs proposed for the survey were based on a rapid scoping review of studies exploring information and communication technology adoption and usage among older adults. Three theoretical models were identified as relevant for this study: (1) the UTAUT [16], (2) the Technology Acceptance Model [18], and (3) the Senior Technology Acceptance Model (STAM [19,20]). A variety of proposed measures were initially circulated in full to HCPs with relevant expertise in NMLD, PPI leads from two NMLD research studies, and the Rare Dementia Support PPI group. Following PPI consultation and feedback relating to length and potential participant burden, scale selection was finalized with the objective of balancing research requirements with participant burden and accessibility for people living with NMLDs. The questionnaire was available in English. The finalized survey scale items are outlined in full in the Table S1 in Multimedia Appendix 1 and described further below.

#### Outcome Measure: Usage Behavior

Usage behavior (UB) was measured using participant-reported technology use (TU) and behavioral intention to use. TU was measured by establishing frequency of use within the 3 months prior to data collection (7-point Likert scale, ranging from “not at all” [0] to “multiple times a day” [6]). Behavioral intention reflected a measure of usage behavior validated in previous work via participant report of 3 questions relating to intention to use technology, responses to which were established using a 10-point Likert scale (“disagree strongly” to “agree strongly” [17]). Usage behavior was calculated as a composite score of TU scores (where 0‐6 frequency scores were rescaled as dummy variables) and behavioral intention to use scores (total score range_BI_: 3‐30), resulting in a total UB score range of 3‐36 reflecting past use frequency and intention to use in the future. Higher scores indicate greater usage behavior of digital resources for health and psychological well-being in the surveyed population. Summing user variables to compute a composite outcome measure and subsequently treating this as a continuous outcome aligns with previous approaches in the literature using the UTAUT2 [17] and STAMs to assess smartphone and technology use in older adults [17,19,23].

#### Factors and Constructs

Predictors comprised constructs validated in the Short STAM 14-item (attitudinal beliefs, n=3; control beliefs, n=4; gerontechnology anxiety, n_items_=2; health, n_items_=5), which has been shown to explain up to 81.5% variance of actual technology usage in older adults [20]. Additional measures of depression and anxiety (Patient Health Questionnaire–4-item [PHQ-4]; [24]), instrumental activities of daily living [25], web-related privacy and security concerns (n_items_=6 [26]) and the Digital Health Care Literacy Scale (n_items_=3 [27]) were added to this survey following consultation of other technology-use studies in clinical older adult cohorts [22] and PPI consultation with NMLD experts-by-experience to further characterize the sample and explore their potential influence on technology use in people with NMLD as well as caregivers and proxy respondents. Likert scales were used for all scale responses, preserving the parameters and scale numbers according to each scale’s administration instructions.

#### Adaptation and Procedure

PPI consultation resulted in several adaptations being incorporated into the final survey. This included using the standardized phrase “web-based resources (eg, Zoom and websites) for your health and psychological well-being” across the scales, replacing varying terms such as “mobile Internet,” “ICT,” and “technology,” to create an accessible term that captured the digital platforms of interest in this study and reduce the number of questions. In addition, online survey branching was introduced to ensure participants would view only the questions relevant to their experience, thus reducing completion time and participant burden.

The finalized scales were uploaded to Qualtrics and replicated in a hard-copy version where preferred. Four survey types were developed to tailor the questions to different possible NMLD experiences:

1. NMLD: for people living with a diagnosis of NMLD;
2. Proxy NMLD: for family members or friends of individuals with NMLD who provided proxy responses;
3. Proxy HCP: for HCPs who provided a proxy response on behalf of a person with NMLD who had capacity to access online resources;
4. Care partners: for family members or friends of those with a diagnosis of NMLD.

All electronic surveys were tested for technical functionality before data collection took place. Surveys were administered to individuals with experience of the following non–memory-led and young-onset dementias: Frontotemporal lobar degeneration (behavioral variant frontotemporal dementia; primary progressive aphasias [semantic variant primary progressive aphasia; nonfluent variant primary progressive aphasia]), atypical Alzheimer disease (posterior cortical atrophy; logopenic variant primary progressive aphasia); dementia with Lewy bodies, inherited dementias (familial frontotemporal dementia; familial Alzheimer disease) and young-onset Alzheimer disease.

### Variable Derivation and Statistical Analysis

Data cleaning and item scoring were undertaken in SAS (SAS Institute) and RStudio (Posit). For analysis purposes, clinical phenotypes were pooled to reflect broad similarities in the presenting symptoms’ cognitive domains. For example, the primary progressive aphasia subtypes (semantic, logopenic, and nonfluent variants, where specified) were subsumed under the label “PPA” to characterize digital health and well-being resource use for individuals with predominant language impairments. Where non–memory-led conditions were either reported to be copresenting (eg, frontotemporal dementia with dementia with Lewy bodies), individuals presented with other syndromes such as young onset Alzheimer disease or vascular dementia, or group sizes precluded condition-specific analyses (eg, individuals living with progressive supranuclear palsy; n=2), these were labeled as “Other.”

Demographic information stratified by survey group is outlined in Table 1. To facilitate comparison across scales, composite scores were normalized, creating *z* scores for outcome and predictor variable responses. To elucidate which factors may be associated with higher usage of digital health and well-being resources, a linear multiple regression was fitted using the UB composite variable as an outcome. Separate multiple regressions were undertaken for each survey type (NMLD, Proxy, HCP, and Carer). To understand whether FTD, PCA, and PPA subtypes were associated with different usage behavior, a robust linear model was undertaken using the UB composite variable as the outcome, with diagnostic category as the main predictor variable and age and gender as covariates. This model was run twice; once collapsing the NMLD and proxy groups and once modeling FTD, PPA, and PCA carers only. Standardized coefficients, 95% CIs, and related significance values (where *P*<.05) are reported for each predictor. The default approach within the lm function in R (R Foundation for Statistical Computing) handles missing data by performing listwise (complete case) deletion. Therefore, cases with any missing data on the predictors were excluded from the regression model. The number of observations for each group is outlined in Multimedia (Table S2 in Multimedia Appendix 1). Acknowledging the smaller sample size, particularly in the NMLD group, model residuals were assessed for normality using statistical tests (Kolmogorov-Smirnov) and visual inspection via Q-Q plots. Testing for multicollinearity by calculating variance inflation factors for variables within each model confirmed that all variance inflation factor values were satisfactory (<5).

**Table 1.** Demographic characteristics and normalized survey scores, split by survey type.

Participant group	NMLD^a^ (N=63)	Proxy NMLD (N=105)	Proxy HCP^b^ (N=101)	Care partners (N=269)
Basic demographic and clinical information				
Age (years), mean (SD)	63.49 (10.54)	67.72 (9.80)	48.79 (11.52)	61.85 (12.88)
Sex (male), n (%)	30 (47.62)	55 (52.38)	77 (76.24)	192 (71.91)
Ethnicity (White), n (%)	62 (100.00)^c^	99 (95.19)	88 (87.13)	251 (93.31)
Time since diagnosis (years), mean (SD)	3.39 (3.05)	3.51 (4.70)	—^d^	3.87 (3.75)
Time since symptoms noticed (years), mean (SD)	5.74 (3.75)	5.97 (3.61)	—	6.81 (4.41)
Dementia subtype, n (%)				
FTD^e^	12 (19.05)	33 (31.43)	17 (16.83)	88 (32.71)
PPA^f^	15 (23.81)	21 (20.00)	67 (66.34)	74 (27.51)
PCA^g^	28 (44.44)	38 (36.19)	2 (1.98)	72 (26.77)
DLB^h^	0 (0.00)	7 (6.67)	1 (0.99)	12 (4.46)
Other	8 (12.70)	6 (5.71)	14 (13.86)	23 (8.55)
Survey completion format, n (%)				
Online	51 (80.95)	99 (94.29)	101 (100.00)	204 (76.40)
Digital health resource use (ever use = yes)	51 (80.95)	55 (52.38)	—	217(80.67)
Scale scores mean raw scores (SD)				
Digital resource use (Composite)	22.81 (10.29)	8.91 (9.20)	13.88 (6.35)	22.16 (9.11)
Attitudinal beliefs	19.84 (8.98)	9.28 (7.22)	21.12 (5.35)	19.66 (7.25)
Control beliefs	27.78 (9.43)	16.68 (8.46)	21.74 (5.75)	32.72 (6.39)
Gerontechnology anxiety	10.28 (6.31)	13.01 (6.83)	12.30 (3.78)	6.26 (5.06)
Health	34.44 (8.88)	25.97 (9.45)	—^d^	35.95 (7.55)
PHQ-4^i^	4.25 (3.16)	5.57 (3.73)	—	3.93 (3.20)
Instrumental activities of daily living	14.88 (7.19)	5.86 (6.34)	—	23.15(2.90)
Online security and privacy concerns	19.28 (10.43)	17.56 (10.32)	20.15 (7.14)	23.04 (11.32)
Digital literacy	5.93 (4.77)	0.89 (2.14)	4.04 (2.52)	10.03 (2.82)

a NMLD: non-memory-led dementia.

b HCP: health care professional.

c Does not reflect total N owing to missing data.

d Not applicable.

e FTD: frontotemporal dementia.

f PPA: primary progressive aphasia.

g PCA: posterior cortical atrophy.

h DLB: dementia with Lewy bodies.

i PHQ-4: Patient Health Questionnaire-4 item.

### Ethical Considerations

Ethical approval was obtained from the University College London (UCL) Research Ethics Committee (8545/004: Rare Dementia Support Impact Study [28]). Potential participants were identified by one of two mechanisms: (1) membership of the UCL-led Rare Dementia Support [29], or (2) membership of the Dementia Research Center participant database. Members of both received email correspondence containing a link to the participant information sheet, online consent form, and survey. To ensure we included individuals who may not use digital health and well-being resources, the e-invitation was supplemented by brief research reminders in several in-person support group meetings, where attendees had the opportunity to read the participant information sheet and ask clarifying questions to the research team, provide written informed consent, and complete a hard copy version of the questionnaire (including a version adapted for posterior cortical atrophy in consultation with a specialist NMLD neuropsychologist).

## Results

**Figure 2.**
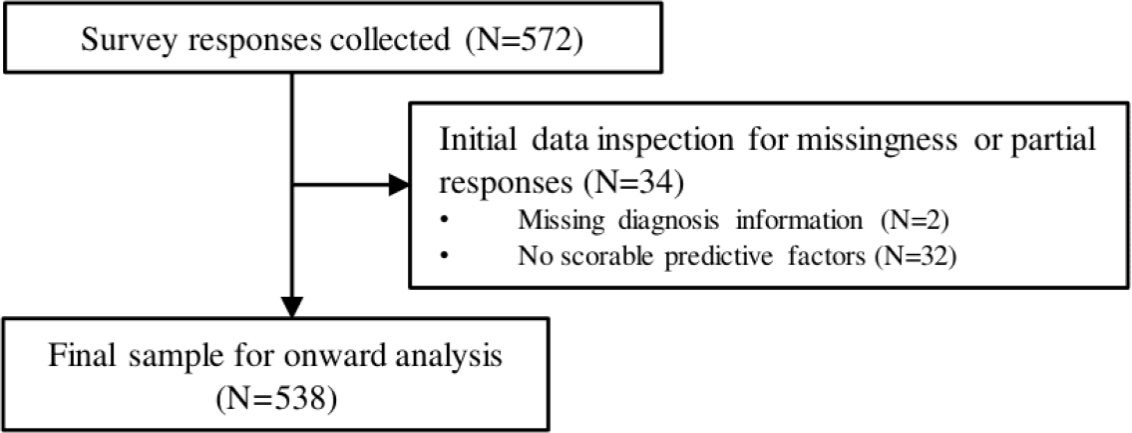
Flowchart depicting response exclusion and final sample selection*.*

The final sample comprised 538 responses from 450 individuals (354 women, 92.9% White; mean age 60.75, SD 13.29 years; see Figure 2). All collected entries were used to calculate the overall survey completion rate (94.1%). A small proportion (0.74%‐3.39%) of data were missing in the remaining survey group datasets, the highest proportion observed for people living with an NMLD response data. Full characteristics of the sample are shown in Table 1, split by NMLD condition and participant group.

Model outputs are available in Table S2 in Multimedia Appendix 1. A higher use of digital resources for health and well-being for NMLD responders was moderately associated with higher attitudinal beliefs (eg, thinking that digital resources might be helpful) (*β*=.43, 95% CI 0.24-0.63; *P*<.001), followed by control beliefs (eg, perceived ease of use; *β*=.30, 95% CI 0.05-0.54; *P*=.02). Adjusted *R*^2^ values indicated the model accounted for 69.3% of the variance in use of health and well-being digital resources.

Proxy NMLD diagnoses were provided in 97.1% of caregiver responses by either a spouse or partner (67.6%) or first-degree relative (parent, child, or sibling; 29.5%). The proxy model output broadly recapitulated the predictors of digital health and well-being resources as reported by individuals living with a diagnosis (attitudinal beliefs *β*=.66, 95% CI 0.52-0.80; *P*<.001; control beliefs *β*=.20, 95% CI 0.05-0.35; *P*=.009). In contrast to the NMLD model, proxy PHQ-4 score was additionally associated with proxy digital health and well-being resource use (*β*=.15, 95% CI 0.03-0.26; *P*=.01), with the overall model accounting for 70.8% of the variance in proxy-reported digital health and well-being resource use.

Responses from HCPs working with people with NMLD demonstrated a range of disciplines, namely NMLD clinicians (neurologists, psychiatrists, and clinical neuropsychologists), nurses, occupational and speech and language therapists, academic researchers, dementia support workers, care home managers, and social workers. One observation in the HCPs’ model, corresponding to an outlier, was identified as deviating from the theoretical normal distribution by approximately 1.0 in the upper tail of the Q-Q plot. No remarkable changes in results were observed when rerunning this model after having excluded this observation, at which point normality assumptions were upheld. The model’s ability to account for the variance of digital health and well-being resource use in the proxy HCPs’ responses was greatly reduced relative to the two previous groups (Adjusted *R*^2^=0.284). Nevertheless, the proxy HCP model was consistent with the findings above in that control beliefs were moderately associated with proxy digital health and well-being resource use (*β*=.38, 95% CI 0.12-0.64; *P*=.005).

Carers responding to the survey about their own use of digital health and well-being resources were consistent with other models in reporting a strong association between digital health and well-being resource use and attitudinal beliefs (*β*=.70, 95% CI 0.61-0.79; *P*<.001). A small association was demonstrated between digital health and well-being resource use and control beliefs (*β*=.13, 95% CI −0.001 to 0.25; *P*=.05). Distinct from previous models, carers’ perception of better health was associated with digital health and well-being resources (*β*=.12, 95% CI 0.02-0.23; *P*=.03). Adjusted *R*^2^ values indicated the model accounted for 63.0% of the variance in use of digital health and well-being resources.

To provide further insight into clinical phenotype associations with digital health and well-being resource use, we conducted exploratory analyses profiling associations of diagnosis (FTD, PPA, and PCA phenotypes) with digital health and well-being resource use. Given the relative concordance between the NMLD and carer-proxy profiles, we considered it appropriate to pool data from these 2 groups and stratify this aggregated group (NMLD+proxy) by clinical phenotype, alongside carer scores for each group (see Table 2). All observations were retained (n=147) for onward analysis. Controlling for covariates age and gender, a robust linear regression indicated that diagnosis category was associated with digital health and well-being resource use for self- and proxy-reported NMLD. Compared to participants with FTD (reference group), higher digital health and well-being resource use were associated with the PCA (*β*=.75, 95% CI 0.33-1.16) and PPA groups (*β*=.55, 95% CI 0.06-1.03). This was recapitulated in the carer groups; higher digital health and well-being resource use was associated with the PCA (*β*=.51, 95% CI 0.23-0.78) and PPA (*β*=.27, 95% CI 0.01-0.54) carer groups relative to FTD carers.

**Table 2.** Demographic, basic survey information, and outcome measure scores depicted for the subgroup of the initial sample, stratified by clinical phenotype of behavioral variant frontotemporal dementia, primary progressive aphasia, and posterior cortical atrophy.

Clinical phenotype	bvFTD^a^	PPA^b^	PCA^c^
Living with NMLD^d^ diagnosis and proxy combined	n=45	n=36	n=66
Age (years), mean (SD)	63.9 (12.4)	71.3 (8.1)	65.4 (8.1)
Female:male (N)	15:30	16:20	42:24
Ethnicity (White), n (%)	43 (95.5)	33 (91.7)	65 (98.5)
Time since diagnosis (years), mean (SD)	3.5 (3.3)	3.1 (1.8)	2.9 (2.6)
Time since symptoms noticed (years), mean (SD)	6.6 (4.2)	5.5 (2.8)	5.3 (3.5)
Online, n (%)	44 (97.8)	31 (86.1)	56 (84.9)
Digital resource use (composite), mean raw score (SD)	9.80 (10.25)	14.44 (12.67)	16.91 (11.34)
Carers	n=88	n=74	n=72
Age (years), mean (SD)	61.5 (13.3)	63.6 (11.3)	59.8 (14.6)
Female:male (N)	75:12^e^	54:20	39:31^e^
Ethnicity (White) n (%)	83 (94.3)	67 (90.5)	68 (94.4)
Time since diagnosis (years), mean (SD)	3.6 (3.2)	4.0 (2.9)	3.5 (2.5)
Time since symptoms noticed (years), mean (SD)	7.3 (4.2)	7.2 (5.5)	5.8 (2.9)
Online, n (%)	67 (76.1)	55 (74.3)	56 (77.8)
Digital resource use (composite), mean raw score (SD)	19.91 (9.02)	22.14 (9.39)	24.10 (8.47)

a bvFTD: behavioral variant frontotemporal dementia.

b PPA: primary progressive aphasia.

c PCA: posterior cortical atrophy.

d NMLD: non-memory-led dementia.

e Does not reflect total N owing to missing data or respondents preferring to self-describe.

## Discussion

### Principal Results

This work was conducted with the aim of understanding the factors associated with digital health and well-being resource use in people with NMLD as well as caregivers and proxy respondents. The findings provide insight for HCPs who seek to maximize engagement with NMLD-specific health and well-being support and underscore considerations for digital NMLD resource design and dissemination. Our findings characterize the extent to which theoretically derived technology use factors associate with digital health and well-being resource use in people with NMLD as well as caregivers and proxy respondents. Here, we report a consistent association between digital health and well-being resource use and control and attitudinal beliefs, regardless of whether it is proxy- or self-reported for people living with NMLDs. Caregiver-proxy reports additionally identified that increased feelings of anxiety and depression in the person with NMLD are associated with their engagement with digital health and well-being resource use. For carers’ own digital health and well-being resource use, perception of better health was an additional predictor of higher use. Carer-reported proxy responses estimated NMLD resource use was lower relative to the reports received directly from individuals with an NMLD diagnosis, and individuals with FTD and their carers demonstrated lower digital health and well-being resource use relative to their PPA and PCA counterparts.

### Interpretations

Controls’ beliefs were positively associated with digital health and well-being resource use across NMLD self-reported and proxy models. Examining the subordinate items within this construct, the likelihood of NMLD engagement with digital health and well-being offering is increased with a platform’s perceived ease of use, the user’s self-efficacy, and facilitating conditions being met (eg, accessibility factors and financial means). Promoting a user’s self-efficacy maximizes the potential of digital platforms for health and well-being. Practically, this finding highlights the importance of training opportunities for people with NMLDs to optimize their experience with any bespoke digital health and well-being resource. This could be achieved by virtue of a tutorial or demonstration by another individual. Training may, for example, involve incorporating initial “onboarding” sessions, which aim to acclimatize the person affected by NMLD to a new digital health and well-being offer. These findings support previous research investigating videoconference-delivered support for people with dementia and their caregivers, whereby integrated technological support and training facilitated ongoing access by participants and users [30-33]. Digital provision of mental health support is available at the time of this writing in the United Kingdom for people with NMLD as well as caregivers if the need of the individuals is deemed to be low in complexity [34]. Evidence suggests that people with NMLD as well as caregivers benefit from accessing such services, therefore underscoring a critical need for NMLD-specific onboarding training as part of this and other digitally mediated mental health provisions [34,35]. In addition, the findings relating to control beliefs highlight the influence of basic accessibility factors in digital health and well-being engagement, which emphasizes the importance of designing for open access for people with NMLD as well as caregivers. Capitalizing on accessibility opportunities, such as working in partnership with community groups and institutions that offer tablet devices and computer access to the general public, will increase equity for individuals affected by NMLD who may otherwise be unable to access specialist digital resources. Taken together, these findings reflect recently reported themes from semistructured interviews exploring technology use in older adults with intellectual disabilities, resulting in recommendations around providing structured, repetitive, and gradual training opportunities for individuals with cognitive challenges, as well as promoting distribution of digital resources in rural areas [36].

Attitudinal beliefs were consistently the strongest predictor of digital health and well-being resources, arguably playing a crucial role in determining whether people living with dementia and their caregivers engage with these resources. This was demonstrated to a greater extent relative to the usability barriers outlined above. This construct reflects the individual user’s perception of these resources being beneficial (eg, enhancing daily effectiveness) and whether they find their use appealing. For people with NMLD as well as caregivers and proxy respondents, the perceived functional value of digital health and well-being resources is critical, perhaps owing to this population seeking digital resources that specifically respond to their unique challenges, which often differ from amnestic dementia experiences. Practically, these findings point to a need for effective communication strategies at the point of offering these resources, which (1) provide a clear scaffold and framing of the digital resource in question and (2) emphasize the positive role that digital resources can play in supporting the navigation of NMLD symptoms, caregiving guidance, or health and well-being support for caregivers.

An additional insight from the NMLD-proxy group indicated that carer-perceived increased digital health and well-being resource use was associated with the person with NMLD’s higher anxiety and depression. Acknowledging that causality may not be inferred from the study design and analysis approach, we venture a range of factors that may explain this finding. For example, depression and anxiety symptoms may be linked to digital health and well-being engagement by virtue of a different profile of help-seeking behavior, either in relation to the person with NMLD consulting the internet for potential mood-related symptom treatment or management through mental health and well-being platforms aimed to target these experiences; or, more broadly, to quell other non–dementia-specific concerns or worries that are exacerbated by virtue of increased anxiety and depression as a result of navigating the challenges dementia brings. Additionally, caregivers providing proxy responses may be aware of their own prompting behaviors, perhaps suggesting that a loved one displaying anxiety and depression may find online resources beneficial for managing their distress. Without further qualitative investigation; however, the mechanisms mediating this association remain speculative.

NMLD caregiver–specific findings indicated that higher self-reported health status predicted higher use of digital health and well-being resources. This differs from previous findings relating to dementia caregiver internet use, whereby caregivers of people primarily with Alzheimer disease or vascular dementia who appraised their own physical health as worse reported significantly more frequent internet use relative to those who appraised their physical health to be similar to a noncaregiver peer of the same age and gender [37]. This contrast may, however, reflect some specificity of the interplay of NMLD caregiver health and digital health resources relative to the same factor’s influence for those caring for individuals with typical dementia variants. This supports the justification for stratifying the needs of these caregivers through bespoke post-diagnostic support and signposting as they form their own distinct profile of factors influencing digital health and well-being technology use.

The distinct cognitive hallmarks of FTD may explain the heterogeneity observed between NMLD phenotypes in relation to digital health and well-being resource use in the self- and proxy-reported group. The most common form of FTD, bvFTD, is characterized by changes in personality and behavior, coupled with early apathy and lack of insight into these symptoms [7]. The lower use of health and well-being digital resources relative to others presenting with different non–memory-led symptoms (as in PCA or PPA) may either reflect a lack of motivation to engage, a failure to even acknowledge or recognize there may be a health problem to consult about, or a combination. Addressing these 2 condition-specific barriers may be key in engaging people with FTD in health and well-being resources related to their condition. For FTD individuals in particular (relative to PPA or PCA), it may be important to explore digital health and well-being resource use design that harnesses methods aiming to increase intrinsic motivation to engage, such as gamification approaches. Unequal diagnostic group sizes resulted in unequal precision as indicated by wider 95% CIs with lower bounds for PPA, consistent with a smaller group size and implying lower sensitivity to detect small effects. We, therefore, interpret this finding with caution. An interesting finding related to the recapitulation of these condition-specific web resource use profiles in the FTD carer groups in relation to the PCA and PPA groups. One potential explanation for this could be reflecting higher care demands specifically in bvFTD, with higher reports of caregiver stress and lower perceived control observed in this caregiver group relative to Alzheimer disease carers [38] and those caring for people with other NMLDs such as PPA [39]. This increase in stress and caregiver burden is likely to diminish FTD carers’ dedicated time and resources to digital use in relation to their own health and well-being needs, in turn reflecting lower use relative to other NMLD caregiver peers.

Taken together, these findings highlight the utmost importance of condition-specific considerations when designing health and well-being digital materials for individuals living with and caring for individuals with NMLDs. However, as a recent systematic review of participatory methods in digital health interventions for dementia caregivers highlighted, subtyping is relatively rare, and many interventions are designed for the typical array of caregiver needs [40]. Approaches to adapt existing support platforms designed with more prevalent dementias in mind (eg, iSupport; World Health Organization) to cater to the specific needs of individuals with NMLDs included several co-design stages to enable iterative changes, including focus groups and validation surveys [41]. Similar approaches have been adopted for the co-development of forthcoming digital health interventions specifically designed by, with, and for specific dementia caregiver subtypes from the outset [13]. Co-design of digital resources and interventions with individuals living with or caring for NMLD is likely critical for ensuring ongoing engagement and interaction with digital provisions designed with people with NMLD as well as caregivers and proxy respondents in mind [42].

### Limitations

Our health care professional model demonstrated far less predictive value of health and well-being digital resource use (only 28% of the variance). This may reflect the instruction given to HCPs to “imagine clients you have worked with,” which does not give ample opportunity for HCPs to share the complexity or breadth of their experience when trying to engage NMLD audiences in digital health and well-being offers. This contrasts with the good explanatory power demonstrated by the NMLD, carer, and proxy models, perhaps reflecting the selective focus of these groups, who were instructed to respond in reference to only one individual (either themselves or one other in the proxy report). Given the HCP group comprised a multidisciplinary group, there is also the potential for differing frames of reference to have impacted model fit. A variability in the professional role within the HCP group, and therefore the clients or service users envisaged when HCP participants completed the survey, may have introduced greater heterogeneity, which in turn would weaken the predictive capacity of the HCP model in contrast to the other models reported here. We acknowledge that the quantitative format of the survey may have also restricted the potential for HCPs to share the depth of expertise that professionals working in this space have to offer. This reflection emphasizes the importance of mixed methods approaches incorporating qualitative inquiry with HCPs to harness the benefits of their range of experiences in this area and to elucidate any discipline-specific observations. This work has been undertaken, and findings are described in a separate publication (Fearn et al, forthcoming).

The definition of technology use in this study was intentionally narrow, concentrating specifically on digital health and well-being resources in order to capture information relating to engagement with NMLD digital offers that are currently available, such as virtual support groups and digital platforms (eg, Rare Dementia Support and Dyscover). This survey did not capture particular devices NMLD users preferred to access these resources, which may be mediated by NMLD audience (persons living with vs carers) or syndromic group and which may have notable influence on whether or not these digital resources are engaged with. FTD populations, for example, exhibit lower smartphone battery usage relative to clinically neurotypical controls [43], which may necessitate other means of presenting health and well-being information for these groups that seem not to engage with smartphone interaction. Together, these findings must also be considered alongside other contextual factors at play, including mediating factors that can influence telehealth success in these individuals, such as the engagement of the communication partner, which has been found to associate with functional outcomes in videoconference-delivered PPA speech and language therapies [44].

Although we endeavored to collect data using both hard-copy paper questionnaires and a complementary digital survey option to capture a broad range of digital access experiences, we acknowledge that the invitations for both formats were distributed electronically. As a result, this sample may overrepresent digitally literate older adults relative to the wider dementia and caregiver populations. Relatedly, internet use is associated most strongly with individuals with dementia specifically from middle-income backgrounds with high educational attainment, characteristics that are also prevalent in membership of specialist support services such as the one from which our sample was derived [45]. Socioeconomic factors similarly associate with internet access in caregiver populations; for instance, individuals with private health insurance are 8 times more likely to have internet access than those without [46]. Furthermore, our sample comprised a very high proportion of White participants, which did not provide the opportunity for exploring potential digital-access patterns and associated factors in more ethnically diverse people with NMLD as well as caregivers and proxy respondents. Our findings and their generalizability should therefore be considered in this context. As awareness for NMLDs grows, there may be opportunities for future research studies to use recruitment strategies that identify participants from more community-based or primary care settings who, in turn, are likely to increase the representativeness relative to this sample. Finally, despite the large overall sample size, we acknowledge that in order to examine group-specific profiles, the number of available observations per group and number of predictors resulted in regressions that may be overfitting. While assumptions were upheld through normality testing with little evidence for multicollinearity, the findings should be interpreted with the understanding that these models are exploratory in nature.

### Conclusions

Collectively, these findings provide insight into several factors that are critical to consider when designing digital health and well-being resources for people with NMLD and their caregivers. The insights from this work underscore the importance of marketing and resource design that resonate with NMLD audiences in terms of targeting practical and emotional perceptions of digital health and well-being offers. This should be undertaken in combination with design considerations that address technical and logistical barriers to access, as well as considering the specific intended audience in terms of specific cognitive and symptom profiles.

## Supplementary material

10.2196/85863 Multimedia Appendix 1 Digital access survey items, full regression model reporting, and Cronbach alpha stratified by survey type.

## Abbreviations

bvFTD: behavioral variant frontotemporal dementia
HCP: health care professional
NMLD: non–memory-led dementia
PCA: posterior cortical atrophy
PHQ-4: Patient Health Questionnaire–4-item
PPA: primary progressive aphasia
PPI: patient and public involvement
Short STAM: Short Senior Technology Acceptance Model
TU: technology use
UB: usage behavior
UCL: University College London
UTAUT: Unified theory of acceptance and use of technology

## References

[1] (2025). Dementia. World Health Organization.

[2] Patel AM, Schuldt R, Boudreau DM, Cobb BR, Win N, McGinley MP (2024). Telemedicine use before and during the COVID-19 pandemic in people with Alzheimer’s disease, multiple sclerosis, or Parkinson’s disease: a cross-sectional study using US commercial claims data. Telemed Rep.

[3] Waddington C, Harding E, Brotherhood EV (2022). The development of videoconference-based support for people living with rare dementias and their carers: protocol for a 3-phase support group evaluation. JMIR Res Protoc.

[4] Lee AR, McDermott O, Orrell M (2023). Understanding barriers and facilitators to online and app activities for people living with dementia and their supporters. J Geriatr Psychiatry Neurol.

[5] Dubois B (2018). The emergence of a new conceptual framework for Alzheimer’s disease. J Alzheimers Dis.

[6] Crutch SJ, Schott JM, Rabinovici GD (2017). Consensus classification of posterior cortical atrophy. Alzheimers Dement.

[7] Rascovsky K, Hodges JR, Knopman D (2011). Sensitivity of revised diagnostic criteria for the behavioural variant of frontotemporal dementia. Brain (Bacau).

[8] Gorno-Tempini ML, Hillis AE, Weintraub S (2011). Classification of primary progressive aphasia and its variants. Neurology (ECronicon).

[9] McKeith IG, Boeve BF, Dickson DW (2017). Diagnosis and management of dementia with lewy bodies: fourth consensus report of the DLB Consortium. Neurology (ECronicon).

[10] Svanberg E, Spector A, Stott J (2011). The impact of young onset dementia on the family: a literature review. Int Psychogeriatr.

[11] Tookey S, Greaves CV, Rohrer JD, Desai R, Stott J (2022). Exploring experiences and needs of spousal carers of people with behavioural variant frontotemporal dementia (bvFTD) including those with familial FTD (fFTD): a qualitative study. BMC Geriatr.

[12] Sullivan MP, Williams V, Grillo A (2022). Peer support for people living with rare or young onset dementia: an integrative review. Dementia (London).

[13] Suárez-González A, John A, Brotherhood E (2023). “Better Living with Non-memory-led Dementia”: protocol for a feasibility randomised controlled trial of a web-based caregiver educational programme. Pilot Feasibility Stud.

[14] Suárez-González A, Zimmermann N, Waddington C (2020). Non-memory led dementias: care in the time of covid-19. BMJ.

[15] Kung CSJ, Steptoe A (2023). Changes in Internet use patterns among older adults in England from before to after the outbreak of the COVID-19 pandemic. Sci Rep.

[16] Venkatesh V, Morris MG, Davis GB, Davis FD (2003). User acceptance of information technology: toward a unified view1. MIS Q.

[17] Venkatesh V, Thong JYL, Xu X (2012). Consumer acceptance and use of information technology: extending the unified theory of acceptance and use of technology1. MIS Q.

[18] Davis FD (1989). Perceived usefulness, perceived ease of use, and user acceptance of information technology. MIS Q.

[19] Chen K, Chan AHS (2014). Gerontechnology acceptance by elderly Hong Kong Chinese: a senior technology acceptance model (STAM). Ergonomics.

[20] Chen K, Lou VWQ (2020). Measuring senior technology acceptance: development of a brief, 14-item scale. Innov Aging.

[21] Ha J, Park HK (2020). Factors affecting the acceptability of technology in health care among older Korean adults with multiple chronic conditions: a cross-sectional study adopting the senior technology acceptance model. Clin Interv Aging.

[22] Harris MT, Rogers WA (2023). Developing a healthcare technology acceptance model (H-TAM) for older adults with hypertension. Ageing Soc.

[23] DeLange Martinez P, Tancredi D, Pavel M, Garcia L, Young HM (2024). Technology acceptance among low-income Asian American older adults: cross-sectional survey analysis. J Med Internet Res.

[24] Kroenke K, Spitzer RL, Williams JBW, Löwe B (2009). An ultra-brief screening scale for anxiety and depression: the PHQ–4. Psychosomatics.

[25] Lawton MP, Brody EM (1969). Assessment of older people: self-maintaining and instrumental activities of daily living. Gerontologist.

[26] Hong W, Thong JYL (2013). Internet privacy concerns: an integrated conceptualization and four empirical studies1. MIS Q.

[27] Nelson LA, Pennings JS, Sommer EC, Popescu F, Barkin SL (2022). A 3-item measure of digital health care literacy: development and validation study. JMIR Form Res.

[28] Brotherhood EV, Stott J, Windle G (2020). Protocol for the rare dementia support impact study: RDS impact. Int J Geriatr Psychiatry.

[29] Advice community learning. Rare Dementia Support.

[30] Austrom MG, Geros KN, Hemmerlein K (2015). Use of a multiparty web based videoconference support group for family caregivers: innovative practice. Dementia (London).

[31] Banbury A, Parkinson L, Gordon S, Wood D (2019). Implementing a peer-support programme by group videoconferencing for isolated carers of people with dementia. J Telemed Telecare.

[32] Loseto-Gerritzen EV, McDermott O, Orrell M (2024). Development of a best practice guidance on online peer support for people with young-onset dementia. Behav Sci (Basel).

[33] Lundberg S (2014). The results from a two-year case study of an information and communication technology support system for family caregivers. Disabil Rehabil Assist Technol.

[34] El Baou C, Saunders R, Buckman JEJ (2024). Effectiveness of psychological therapies for depression and anxiety in atypical dementia. Alzheimers Dement.

[35] Lattie EG, Stiles-Shields C, Graham AK (2022). An overview of and recommendations for more accessible digital mental health services. Nat Rev Psychol.

[36] Álvarez-Aguado I, Vega Córdova V, Muñoz La Rivera F (2025). Exploring technology use among older adults with intellectual disabilities: barriers, opportunities, and the role of advanced technologies. Disabil Rehabil Assist Technol.

[37] Teles S, Paúl C, Costa-Santos C, Ferreira A (2022). Use of dementia and caregiving-related internet resources by informal caregivers: a cross-sectional study. Front Med (Lausanne).

[38] Wong C, Merrilees J, Ketelle R, Barton C, Wallhagen M, Miller B (2012). The experience of caregiving: differences between behavioral variant of frontotemporal dementia and Alzheimer disease. Am J Geriatr Psychiatry.

[39] Mioshi E, Foxe D, Leslie F (2013). The impact of dementia severity on caregiver burden in frontotemporal dementia and Alzheimer disease. Alzheimer Dis Assoc Disord.

[40] Messina A, Annoni AM, Amati R (2025). Participatory methods in designing digital health interventions for informal caregivers of people with dementia. A systematic review. Internet Interv.

[41] Naunton Morgan B, Windle G, Lamers C, Brotherhood E, Crutch S, Rare Dementia Support (RDS) Impact Project Research Team (2023). Adaptation of an eHealth intervention: iSupport for carers of people with rare dementias. Int J Environ Res Public Health.

[42] Talbot CV, Briggs P (2022). The use of digital technologies by people with mild-to-moderate dementia during the COVID-19 pandemic: a positive technology perspective. Dementia (London).

[43] Paolillo EW, Casaletto KB, Clark AL (2024). Examining associations between smartphone use and clinical severity in frontotemporal dementia: proof-of-concept study. JMIR Aging.

[44] Rogalski E, Roberts A, Salley E (2022). Communication partner engagement: a relevant factor for functional outcomes in speech-language therapy for aphasic dementia. J Gerontol B Psychol Sci Soc Sci.

[45] Nakagomi A, Kondo K, Shiba K (2025). Heterogeneity in the association between internet use and dementia among older adults: a machine-learning analysis. Arch Gerontol Geriatr.

[46] Cameron E, Mansfield E, Ampofo A, Coda A, Boyes A (2025). Internet access and use among dementia carers and the people they support in Australia: cross-sectional survey. JMIR Form Res.

