# Supplementary material for: Factors Associated With the Use of Digital Health and Well-Being Resources in Non–Memory-Led Dementias: Quantitative Survey Study
Source: JMIR Aging. 2026 Mar 30;9:e85863. doi: 10.2196/85863 (PMC13035076; doi:10.2196/85863)
Supplement: Multimedia Appendix 1 [file aging-v9-e85863-s001.docx]

**Supplementary materials: Digital access survey items, full regression model reporting and Cronbach alpha stratified by survey type.**

Table 1. Variables and constructs collected in the digital access survey for nonmemory-led and genetic dementias

| Model [Source] | Superordinate construct | Conceptual subscale | Item label | Items (adapted for survey) | Response options |
| --- | --- | --- | --- | --- | --- |
| UTAUT2 | Technology Use  [TU] |  | TU1 | In the last three months, how often have you used web-based resources (e.g. Zoom, websites) for your health and wellbeing? | Not at all –  Multiple times a day |
|  | Behavioural intention  [BI] |  | BI1 | You intend to [continue to]^b^ use web-based resources (e.g., Zoom, websites) for your health and psychological wellbeing. | 1 [Strongly disagree] –  10 [Strongly agree] |
|  |  |  | BI2 | You will always try and use web-based resources (e.g., Zoom, websites) for your health and psychological wellbeing in your daily life. |  |
|  |  |  | BI3 | You plan to [continue to]^2^ use web-based resources (e.g., Zoom, websites) for your health and psychological wellbeing frequently. |  |
| Short STAM[20] | Attitudinal beliefs  [ATT] | Perceived usefulness  [PU] | PU1 | Using web-based resources (e.g., Zoom, websites) for your health and psychological wellbeing would enhance your effectiveness in daily activities. |  |
|  |  |  | PU3 | You would find web-based resources for your health and psychological wellbeing useful in your daily activities. |  |
|  |  | Attitudinal beliefs  [ATT] | ATT2 | You like the idea of using web-based resources for your health and psychological wellbeing. |  |
|  | Control beliefs  [CB] | Perceived ease of use  [PEOU] | PEOU2 | You could be skillful at using web-based resources for your health and psychological wellbeing. |  |
|  |  | Gerontechnology self-efficacy  [SE] | SE1 | You could complete a task using web-based resources for your health and psychological wellbeing if there is someone to demonstrate how. |  |
|  |  | Facilitating conditions  [FC] | FC3 | Your financial status does not limit your activities in using web-based resources for your health and psychological wellbeing. |  |
|  |  |  | FC4 | When you want or need to use web-based resources for your health and psychological wellbeing, they are accessible for you. |  |
|  | Gerontechnology anxiety  [ANX] | - | ANX1 | You feel apprehensive about using web-based resources for your health and psychological wellbeing. |  |
|  |  |  | ANX2 | You hesitate to use web-based resources for your health and psychological wellbeing for fear of making mistakes you cannot correct. |  |
|  | Health  [H]^3^ | Self-reported health conditions  [H] | H1 | How is your general health? | 1 [Poor] –  10 [Excellent] |
|  |  | Cognitive ability [C] | C3 | How well are you able to concentrate? |  |
|  |  | Social relationships  [S] | S1 | How satisfied are you with your personal relationships? | 1 [Extremely unsatisfied] –  10 [Extremely satisfied] |
|  |  |  | S2 | How satisfied are you with the support you get from your friends and family? |  |
|  |  | Attitude to ageing and life satisfaction (A) | A2 | How satisfied are you with your quality of life? |  |
| [24] | Anxiety and Depression  [PHQ-4]^3^ | - | PHQ-4 [1] | Feeling nervous, anxious, or on edge. | Not at all –  Nearly everyday |
|  |  |  | PHQ-4 [2] | Not being able to stop or control worrying. |  |
|  |  |  | PHQ-4 [3] | Feeling down, depressed, or hopeless. |  |
|  |  |  | PHQ-4 [4] | Little interest or pleasure in doing things. |  |
| [25] | Instrumental activities of daily living  [IADL]^3^ | - | IADL1 | How would you rate your ability to:  Use the telephone independently? | Independently –  By others |
|  |  |  | IADL2 | Do your grocery shopping independently? |  |
|  |  |  | IADL3 | Prepare your food independently? |  |
|  |  |  | IADL4 | Do housework or handyman work independently? |  |
|  |  |  | IADL5 | Do your laundry independently? |  |
|  |  |  | IADL6 | Get to places beyond walking distance independently? |  |
|  |  |  | IADL7 | Take medications independently? |  |
|  |  |  | IADL8 | Manage money independently? |  |
| [26] | Health Information Privacy Concerns  [HIPC] | Collection  [COL] | COL3 | I am concerned that healthcare providers are collecting too much personal health information about me. | 1 [Disagree strongly] –  7 [Agree strongly] |
|  |  | Secondary Usage  [SEC] | SEC3 | I am concerned that healthcare providers would share my personal health information with other healthcare organisations without my authorisation. |  |
|  |  | Errors  [ERR] | ERR2 | I am concerned that healthcare providers do not have adequate procedures to correct errors in my personal information. |  |
|  |  | Improper Access  [ACC] | ACC2 | I am concerned that healthcare providers do not devote enough time and effort to preventing unauthorised access to my personal health information. |  |
|  |  | Control  [CON] | CON2 | It usually bothers me when I do not have control or autonomy over decisions about how my personal health information is used and shared by healthcare providers. |  |
|  |  | Awareness  [AWA] | AWA2 | It usually bothers me when I am not aware or knowledgeable about how my personal health information will be used by healthcare providers. |  |
| [27] | Digital health literacy  [DHLS] |  | DHLS1 | I can use applications/programmes (like Zoom) on my mobile phone, computer, or another electronic device on my own (without asking for help from someone else). | 0 [Strongly disagree] –  4 [Strongly agree] |
|  |  |  | DHLS2 | I can set up a video chat using my mobile phone, computer, or another electronic device on my own (without asking for help from someone else). |  |
|  |  |  | DHLS3 | I can solve or figure out how to solve basic technical issues on my own (without asking for help from someone else). |  |

^1^ Phrasing was adapted for proxy responses to reflect question was posed in relation to the person living with NMLD. ^2^ Phrasing was adapted as indicated to reflect whether participant response outlined current or no use of web-based resources for health and wellbeing; ^3^Not administered in the healthcare professional proxy surveys. ^a^ For carer proxy surveys, respondents were asked to answer these questions in relation to ‘their loved ones using web-based resources for their health and psychological wellbeing’. ^b^ For healthcare professionals, respondents were asked to answer these questions in relation to ‘people living with a rare dementia’.

20. Chen K, Lou VWQ. Measuring senior technology acceptance: development of a brief, 14-item scale. Innov Aging. 2020;4(3):igaa016. [doi: 10.1093/geroni/igaa016] [Medline: 32617418]

24. Kroenke K, Spitzer RL, Williams JBW, Löwe B. An ultra-brief screening scale for anxiety and depression: the PHQ–4. Psychosomatics. Nov 2009;50(6):613-621. [doi: 10.1016/S0033-3182(09)70864-3]

25. Lawton MP, Brody EM. Assessment of older people: self-maintaining and instrumental activities of daily living. Gerontologist. 1969 Autumn;9(3):179-86. PMID: 5349366.

26. Hong W, Thong JYL. Internet privacy concerns: an integrated conceptualization and four empirical studies1. MIS Q. Mar 1, 2013;37(1):275-298. [doi: 10.25300/MISQ/2013/37.1.12]

27. Nelson LA, Pennings JS, Sommer EC, Popescu F, Barkin SL. A 3-item measure of digital health care literacy: development and validation study. JMIR Form Res. Apr 29, 2022;6(4):e36043. [doi: 10.2196/36043] [Medline: 35486413]

Table 2. Multiple regression models of digital health and wellbeing resource usage behaviour.

|  | **NMLD**  N(obs) = 49 | **Proxy NMLD**  N(obs) = 92 | **Proxy HCP**  N(obs) = 97 | **Carer**  N(obs) = 236 |
| --- | --- | --- | --- | --- |
|  | Standardised beta coefficients [95% CI], *P* value | | | |
| Intercept | **.26 [.08, .43]**  ***P* = .0076** | -.06 [-.35, .23]  *P* = .677 | .04 [-.19, .26]  *P* = .759 | .02 [-.13, .17]  *P* = .814 |
| Attitudinal beliefs | **.43 [.24, .63]**  ***P* < .001** | **.66 [.52, .80]**  ***P* < .001** | .14 [-.07, .36]  *P* = .190 | **.70 [.61, .79]**  ***P* < .001** |
| Control beliefs | **.30 [.05, .54]**  ***P* = .018** | **.20 [.05, .35]**  ***P = .*009** | **.38 [.12, .64]**  ***P* = .005** | .13 [-.001, .25]  *P* = .051 |
| Gerontechnology anxiety | .05 [-.13, .22]  *P* = .604 | .06 [-.03, .16]  *P* = .190 | -.18 [-.39, .04]  *P* = .102 | .02 [-.07, .12]  *P* = .656 |
| Health | .12 [-.14, .37]  *P* = .356 | .12 [-.01, .25]  *P* = .070 | - | **.12 [.02, .23]**  ***P* = .026** |
| PHQ-4 | .05 [-.17, .26]  *P* = .652 | **.15 [.03, .26]**  ***P* = .011** | - | .04 [-.05, .13]  *P* = .330 |
| Independence of daily living | .11 [-.24, .45]  *P* = .535 | .11 [-.07, .29]  *P* = .243 | - | .004 [-.21, .22]  *P* = .972 |
| Digital privacy concerns | -.08 [-.24, .10]  *P* = .382 | -.05 [-.16, .05]  *P* = .300 | .06 [-.12, .25]  *P* = .498 | .06 [-.01, .12]  *P* = .086 |
| Digital health literacy | .7 [-.25, .39]  *P* = .668 | -.13 [-.44, .18]  *P* = .408 | .20 [-.07, .47]  *P* = .138 | .06 [-.09, .20]  *P* = .451 |
| Adjusted R^2^ | 0.693 | 0.708 | 0.284 | 0.630 |

Separate multiple regressions were run for each survey type, the multiple regression model included the UB composite as an outcome. Standardised beta coefficients [95% CI] relating to model output are listed. Bold indicates p < 0.05. – indicates these scales were not administered.

Table 3. Cronbach’s Alpha reported for the current sample.

|  |  | | | **NMLD** | **Proxy NMLD** | **Proxy HCP** | **Carer** | |
| --- | --- | --- | --- | --- | --- | --- | --- | --- |
|  | | *N* items | Cronbach’s α | | | | |  |
| Attitudinal beliefs (ATT) | | 3 | 0.94 | | 0.91 | 0.90 | 0.95 |  |
| Control beliefs (CON) | | 4 | 0.76 | | 0.70 | 0.58 | 0.64 |  |
| Gerontechnology anxiety (ANX)* | | 2 | 0.86 | | 0.91 | 0.80 | 0.87 |  |
| Health | | 5 | 0.81 | | 0.82 | - | 0.77 |  |
| Anxiety and depression (PHQ-4) | | 4 | 0.84 | | 0.83 | - | 0.87 |  |
| Independence of daily living (ADL) | | 8 | 0.89 | | 0.91 | - | 0.92 |  |
| Digital privacy concerns (PRIV) | | 6 | 0.91 | | 0.96 | 0.89 | 0.94 |  |
| Digital health literacy (DHL) | | 3 | 0.94 | | 0.87 | 0.89 | 0.87 |  |

* For two‑item scales (e.g. ANX), internal consistency was estimated using the Spearman–Brown prophecy formula. – indicates scale not administered.
